# SUPPORTING AGING IN PLACE WITH THE INTERNET OF THINGS: MEETING CHALLENGES OF USE THROUGH AUGMENTED REALITY TOOLS

**DOI:** 10.1093/geroni/igz038.2789

**Published:** 2019-11-08

**Authors:** Laura Levy, Maribeth Gandy

**Affiliations:** Georgia Institute of Technolopgy, Atlanta, Georgia, United States

## Abstract

Internet of Things (IoT) devices (including smart thermostats, lightbulbs, and door locks) have the potential to greatly enhance independence and promote aging-in-place among older adults with mobility disabilities. However, these devices require extensive information technology expertise to select, configure, use, and adapt to meet one’s needs and this creates considerable barriers to their adoption, acceptance, and utilization. Meanwhile, increasingly available consumer augmented reality (AR) technologies can enable individuals to experience how IoT function and are used within the context of one’s own abilities and home. This may provide potential users an effective means of overcoming barriers to adoption, acceptance, and utilization of IoT to support aging in place. We present preliminary results from a participatory design study of older adults with mobility disabilities on the use of AR on a smart phone device to support IoT understanding.

